# Adrenal mass in a patient with tetralogy of Fallot: beyond expected 

**DOI:** 10.15171/jcvtr.2016.38

**Published:** 2016-12-30

**Authors:** Efrén Martínez-Quintana, Fayna Rodríguez-González

**Affiliations:** ^1^Cardiology Service, Insular-Materno Infantil University Hospital, University of Las Palmas de Gran Canaria, Las Palmas de Gran Canaria, Spain; ^2^Dr. Negrín University Hospital of Gran Canaria, Las Palmas de Gran Canaria, Spain

## Dear Editor,

We have read with interest the article published by Tapia-Orihuela et al.^1^ However, in the wake of that reading, we would like to make a few comments that we believe to be important.

Firstly, severe pulmonary arterial hypertension is an infrequent finding in patients with tetralogy of Fallot an if it exists it is usually in the context of previous palliative operations, major aortopulmonary collateral arteries or left heart failure. The aim of palliative procedures for congenital heart defects (CHD) is to lessen cyanosis or prepare the circulation for later correction. However, central shunts (Potts, Waterston-Cooley and classic Blalock-Taussig’s shunts) have been abandoned due to the high incidence of complications including pulmonary arterial hypertension. We do not know what type of palliative surgery was done to the reported patient. However, it is well known that patients with Eisenmenger physiology have better survival rates than patients with primary pulmonary hypertension who have an intact ventricular septum.^2^ For this reason repair must be avoided in such CHD patients.

Secondly, significant pulmonary regurgitation is almost always encountered following a transannular patch repair and although it is usually well tolerated for years it finally leads to symptomatic right ventricular dilation and dysfunction moreover if it coexists with significant tricuspid regurgitation. Both valvular pathologies are suspected, although pulmonary regurgitation is not mentioned, in the patient reported by Tapia-Orihuela et al^1^ according to their echocardiographic and auscultatory data. When valvular regurgitations are severe, pulmonary valve replacement and tricuspid annuloplasty should be performed before irreversible right ventricle dysfunction ensues.^3^ The deleterious long-term effects of pulmonary regurgitation increases with co-existing distal pulmonary artery stenosis or pulmonary arterial hypertension as ocurres in the reported patient.

Thirdly, hepatic complications are common in CHD patients and may occur secondary to persistent chronic passive venous congestion, decreased cardiac output, transfusion or drugs. When cardiac cirrhosis is established any hepatic mass should be studied and followed up to discard de presence of hepatocellular carcinoma. Also, patients with lower oxygen saturation use to have higher hemoglobin concentration. For this reason, in this patient, we should rule out anemia of chronic disease or bleeding in the context of portal hypertension. Similarly, the differential diagnosis of any adrenal mass should include not only pheochromocytoma but also other primary tumors. Taken into account that computed tomography may not differentiate between some of these tumors and that stress situations, as seen in their Fallot patient, may by itself elevate blood and urine metanephrine levels we should perform a biopsy to establish the histological diagnosis. Moreover, having cortisol levels within normal range dose not discard having an adrenal carcinoma^3^ as previously reported in Fallot patients.^4^

Finally, people living at high altitude are at increased risk for pheochromocytomas and paragangliomas as stated by the authors. Therefore, hypoxia in CHD patients may be a compensatory hyperplasic mechanism turning adrenal medular hyperplasia into an autonomously functioning medular tumor. However, although the vast majority of congenital heart disease patients use to have a history of long-standing cyanosis many had undergone biventricular repair or had been converted to a Fontan circulation long before the diagnosis of the pheochromocytomas/ paragangliomas and were not hypoxemic at the time of diagnosis.^5^

## Ethical issues

Not applicable.

## Competing interests

Authors declare no conflict of interest in this study.
